# Association of Pre-Disease Body Mass Index With Multiple Sclerosis Prognosis

**DOI:** 10.3389/fneur.2018.00232

**Published:** 2018-05-11

**Authors:** Ali Manouchehrinia, Anna Karin Hedström, Lars Alfredsson, Tomas Olsson, Jan Hillert, Ryan Ramanujam

**Affiliations:** ^1^Department of Clinical Neuroscience, Karolinska Institutet, Karolinska University Hospital Solna, Stockholm, Sweden; ^2^Unit of Cardiovascular Epidemiology, Institute of Environmental Medicine, Karolinska Institutet, Stockholm, Sweden; ^3^Centre for Occupational and Environmental Medicine, Stockholm County Council, Stockholm, Sweden; ^4^Department of Mathematics, The Royal Institute of Technology, Stockholm, Sweden

**Keywords:** body mass index, progression, disability, secondary progressive MS, multiple sclerosis

## Abstract

Both high body mass index (BMI) and smoking tobacco are known risk factors for developing multiple sclerosis (MS). However, it is unclear whether BMI, like smoking, is a risk factor for the secondary progressive (SP) course. We, therefore, sought to determine if high/low BMI at age 20 is associated to risk of SP development, in the context of smoking status. Using data from MS patients with BMI and smoking information available, we examined relapsing onset patients with MS onset after 20 years of age. Cox regressions were conducted on smokers and non-smokers, with BMI as the main exposure. In total, 5,598 relapsing onset MS patients were included. The models demonstrated that BMI > 30 was associated to increased risk of SPMS in smokers (hazard ratio 1.50, *p* = 0.036). This association of obesity at age 20 with increased risk of SP was not observed in non-smokers (hazard rate 0.97, *p* = 0.900). Since the risk is confined to smokers, the interaction observed may give insight to disease driving mechanisms.

## Introduction

Multiple sclerosis (MS) is a chronic inflammatory disorder, which affects the central nervous system, characterized by degradation of myelin and axonal loss. It is a complex neurological disorder, in which genes and environmental factors act together to predispose individuals to the disease. Occurring in approximately 189 of 100,000 individuals in Sweden (1), MS most frequently occurs in bouts wherein symptoms occur during relapses, followed by periods during which the patient typically experiences a reduction of symptoms. Most such relapsing-remitting patients experience a change to continuous worsening of symptoms termed secondary progressive (SP) MS. The time to this event is an important measure of disease progression, and median time is 23 years after disease onset (2). Increasing the time to SP, as well as lessening relapse rates, is a goal of treatment.

A genetic role in MS has been well documented, with the HLA-DRB1 and HLA-A2 alleles being the strongest factors influencing risk. Genome-wide association studies have found a large number of genes associated with MS, with modest odds ratios (3, 4). Several environmental factors have been associated with MS development including vitamin D and sun exposure, night shift work, Epstein–Barr virus positivity, and smoking (5). Increased body mass index (BMI) in childhood and adolescence is another reported risk factor for MS development (6–8). However, when it comes to factors affecting the progression of MS as defined by conversion to SPMS, very few have been identified. In fact, apart from demographic factors such as age (9), sex (2), and socioeconomic status (10), the main identified factors are age at onset, comorbidity (11), exposure to disease modifying therapy (12), and cigarette smoking (13–16). Progress made to identify additional factors associated to MS progression will have to rely on aggregate data such as the large patient repository available in the Swedish MS Registry (SMSreg) and related studies, which contain the necessary clinical, environmental, and lifestyle information. A previous study made use of a smaller subset of data (*n* = 728) to report a risk of continued cigarette smoking after disease diagnosis on the time to SPMS disease in smokers using an accelerated time to failure model (1.047 acceleration factor, *p* < 0.001) (17). Here, we utilize the same set of resources with a much large patient pool to address a related issue, namely whether patient BMI outside the normal range affects MS progression. However, changes in BMI may be correlated to smoking status and quantity of smoking, which could affect the results of this and previous studies. We, therefore, sought to determine if patient BMI at age 20, and before disease onset, is associated to SP risk in a cohort of substantial numbers for which information on smoking status was available.

## Materials and Methods

Data were obtained from the Genes and Environment in Multiple Sclerosis (GEMS) (18) study of prevalent MS patients. They were identified from the SMSreg and diagnosed according to the McDonald criteria. Questionnaires were collected from patients including information on smoking habits, BMI, and other factors between November 2009 and November 2011.

In the SMSreg, SPMS is retrospectively diagnosed by the practicing neurologist according to the Lublin 1996 criteria during yearly annual clinic follow-up. SPMS in Sweden is defined as progression of disability with or without occasional relapses, minor remissions, and plateaus. Age at the onset is the age at the first clinical symptom suggestive of MS which was identified and recorded by a MS specialist neurologist.

Additionally, patient data from the EIMS (Epidemiological Investigation of Multiple Sclerosis) were collected as part of an incident study of MS patients in Sweden. EIMS comprised the population aged 16–70 years in Sweden. Incident cases were recruited *via* 40 study centers, including all university hospitals in Sweden. All cases were examined and diagnosed by a neurologist located at the unit where the case was entered. This study also included patient questionnaires pertaining to smoking and BMI, and was previously described (19). The participation rate was 92% in EIMS and 82% in GEMS. Both studies were approved by the Stockholm Region Ethical Review Board.

Patients self-reported being an ever or never smoker before disease onset. BMI at the age of 20 was calculated using height and weight as self-reported from memory, and current BMI was also provided. All patient data from the GEMS and EIMS were used that included BMI at age 20, smoking status before onset, age at onset as recorded in the SMSreg, sex, and SP status at censor date (SMSreg).

The date of conversion to SPMS was estimated by the treating neurologist, typically in conjunction with a clinical visit, using the established SP definition. Individuals were removed who had documented MS onset before 20 years of age since BMI at age 20 would otherwise be obtained inside the measurement interval.

This study was approved by the Stockholm regional ethical committee at Karolinska Institutet.

### Statistical Analyses

Body mass index was grouped according to the standard BMI categories defined by the World Health Organization, namely BMI < 18.5 being underweight, BMI between 18.5 and 24.9 being normal, BMI between 25 and 29.9 being overweight and BMI > 30 being obese (20). For purposes of investigating the effects at extreme BMI, a combined BMI range of 18.5–30 was used as the reference. Numbers of patients with BMI at age 20 corresponding to these categories were compiled to determine if any differences exist between men/women and smokers/non-smokers, a possible explanation for BMI variation between them.

Uncorrected Kaplan–Meier plots were created for the entire cohort, as well as for the BMI strata, in order to visualize the median time to conversion among patients.

Cox proportional hazard models were used to measure the risk of each variable on the development of SPMS, using age at onset for entry time as and age at censor (date of conversion to SP or last clinical visit if still relapsing-remitting) as exit time. Separate models were created for smokers and non-smokers in order to separate effects of BMI categories from smoking risk. In order to determine if either very low or high BMI were risk factors, BMI levels according to low (<18.5) and obese (>30) were used with (18.5–30) as reference. Patient sex was also included as a covariate. Statistical significance was defined at the 0.05 level.

Models were checked for the proportionality of hazard using weighted residuals (21). All statistical analyses and survival models were created in R version 3.3.2 (22).

## Results

From the total of 7,672 patients in the dataset 756 patients with primary progressive or unknown disease course, 567 patients with onset ≤20 years, and 751 patients with erroneous or incomplete data were excluded. In total, 5,598 relapsing onset patients were included in this study, 4,145 women and 1,453 men. Basic clinical and demographic characteristics for study participants are given in Table 1. The distribution of men and women across smoking categories and BMI is given in Table 2. The median pack years smoked before onset for BMI < 18.5 was 7.00, compared to 5.50 in the normal BMI group, 5.95 in the overweight group, and 4.93 in the obese group. Proportions of men and women in each smoking category did not significantly vary for each BMI strata.

**Table 1:** Basic clinical and demographic comparisons of patients in BMI groups.

	BMI categories			
	≤18.5	18.5–30	>30	*p*-Value
Number	668	4,772	158	
Age at last clinic assessment [mean (SD)]	51 (11.19)	48 (11.08)	42 (10.12)	<0.001
Onset age [mean (SD)]	35 (9.53)	34 (8.95)	30 (6.99)	<0.001
Follow-up time [mean (SD)]	15 (10.03)	14 (9.45)	11 (8.35)	<0.001
Calendar year of birth [mean (SD)]	1956 (11.89)	1960 (12.23)	1967 (10.85)	<0.001
Calendar year of onset [mean (SD)]	1991 (11.74)	1994 (11.07)	1998 (9.14)	<0.001
Sex (female %)	609 (91.2)	3,421 (71.7)	115 (72.8)	<0.001
Transited to secondary progression (%)	295 (44.2)	1,663 (34.8)	43 (27.2)	<0.001
Used smokeless tobacco (%)	16 (2.4)	532 (11.1)	21 (13.3)	<0.001
Smoking (ever smoked %)	431 (64.5)	2,757 (57.8)	94 (59.5)	0.004
Ever treated with immunomodulatory treatments (%)	464 (69.5)	3,673 (77.0)	133 (84.2)	<0.001
Duration of treatment [years, mean (SD)]	5.47 (3.88)	5.37 (3.52)	5.41 (2.76)	0.947

*BMI, body mass index*.

**Table 2:** Distribution of patients across BMI categories for smokers and non-smokers.

	Non-smokers (%)		Smokers (%)	
**Women (***n*** = 4,145)**				
BMI < 18.5	144	8.26	282	11.74
BMI 18.5–24.9	1,402	80.44	1,858	77.35
BMI 25–29.9	150	8.61	194	8.08
BMI > 30	47	2.70	68	2.83
Total	1,743		2,402	
**Men (***n*** = 1,453)**				
BMI < 18.5	14	2.44	25	2.84
BMI 18.5–24.9	457	79.76	707	80.34
BMI 25–29.9	85	14.83	122	13.86
BMI > 30	17	2.97	26	2.95
Total	573		880	

*BMI, body mass index*.

Table 2 indicates that there were slightly more underweight female smokers than non-smokers (11.74 vs. 8.26%), while this proportion was similar in men. Although low BMI smokers had slightly higher pack years before onset (7.0 compared with 4.9–6.0 in other BMI groups), pack years smoked was not statistically significant in the survival model.

**Figure 1:**
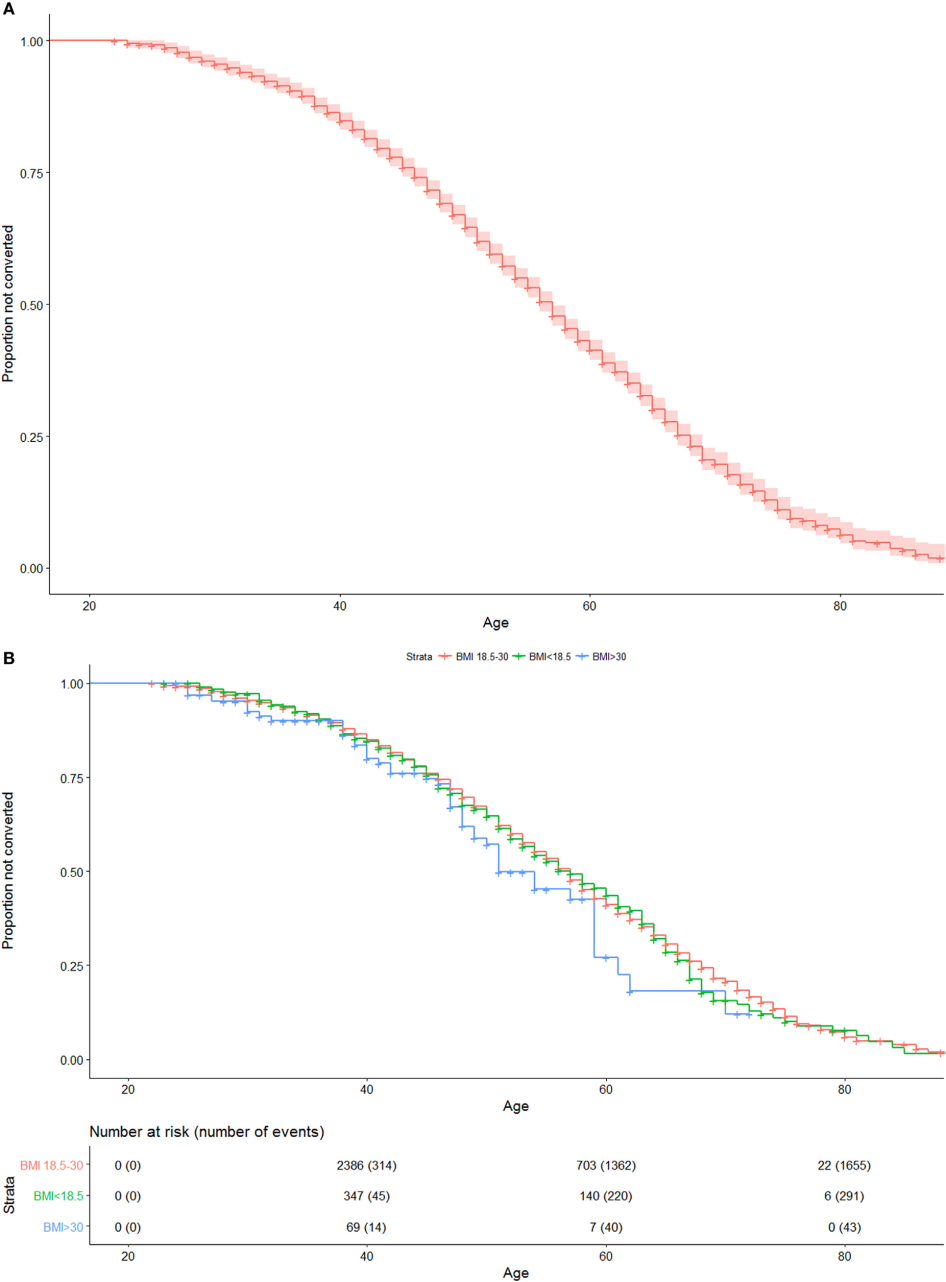
**(A)** Uncorrected Kaplan–Meier plot of conversion to SPMS of entire cohort. Numbers at risk and cumulative numbers of events are presented. **(B)** Uncorrected Kaplan–Meier plot of conversion to SPMS of entire cohort, when stratified by body mass index (<18.5, 18.5–30, >30). Numbers at risk and cumulative numbers of events are presented.

Median follow-up time was 12 years (interquartile range 7 to 19). An uncorrected Kaplan–Meier plot of all patients included in the study is shown in Figure 1A, with median age at SPMS for the entire cohort being 57 years. Figure 1B shows an uncorrected Kaplan–Meier plot of all patients when stratified by BMI, indicating (not statistically significant) that patients who were obese at age 20 experience earlier median age at conversion to SPMS [51 (95%CI: 49–59) years compared with 57 (95%CI: 56–57) years in other BMI groups].

Cox regressions for smokers and non-smokers were conducted independently, with results given in Table 3. Obesity (BMI > 30) was significantly associated with SP in the smoking group (Hazard ratio 1.50, *p* = 0.036). Male sex was significantly associated to SP in both models.

**Table 3:** Cox regression summary for smokers (*n* = 3,282, 1,227 events) and non-smokers (*n* = 2,316, 772 events).

	Hazard ratio	Lower CI	Upper CI	*p*-Value
**Non-smokers before onset**				
Sex (men as reference)	0.72	0.60	0.84	<0.001*
BMI categories				
≤18.5	1.11	0.90	1.37	0.310
18.5–30 reference	Ref.	Ref.	Ref.	Ref.
>30	0.97	0.57	1.61	0.900
Smokeless tobacco use	1.25	1.03	1.50	0.018*
**Smokers before onset**				
Sex (men as reference)	0.79	0.69	0.89	<0.001*
BMI categories				
≤18.5	1.10	0.94	1.29	0.234
18.5–30 reference	Ref.	Ref.	Ref.	Ref.
>30	1.50	1.03	2.18	0.036*
Smokeless tobacco use	1.01	0.88	1.15	0.901

*BMI, body mass index; CI, confidence intervals*.

*Significant p-Values.

Finally, a set of models examined whether an interaction between BMI at age 20 and sex was present. As shown in Table 4, no significant interaction between sex and BMI category was present for either the smoker or non-smoker groups.

**Table 4:** Cox regression summary for smokers (*n* = 3,282, 1,227 events) and non-smokers (*n* = 2,316, 772 events), with interaction present.

	Hazard ratio	Lower CI	Upper CI	*p*-Value
**Non-smokers before onset**				
Sex (men as reference)	0.71	0.60	0.84	<0.001*
BMI categories				
<18.5	0.96	0.49	1.88	0.895
18.5–30, reference				
≥30	1.01	0.44	2.29	0.987
Smokeless tobacco use	1.25	1.03	1.50	0.021*
Sex (female)* BMI category				
<18.5	1.19	0.58	2.42	0.636
≥30	0.94	0.33	2.69	0.902
**Smokers before onset**				
Sex (men as reference)	0.81	0.70	0.93	0.002*
BMI categories				
<18.5	1.27	0.84	1.92	0.258
18.5–30, reference				
≥30	1.92	1.10	3.36	0.021*
Smokeless tobacco use	1.02	0.89	1.16	0.829
Sex (female)* BMI category				
<18.5	0.85	0.54	1.33	0.464
≥30	0.66	0.31	1.40	0.271

*An interaction between sex (female) and BMI categories (<18.5 and ≥30) is presented*.

*BMI, body mass index. CI, confidence intervals*.

*Significant p-Values.

## Discussion

The risk of increased BMI in youth for developing MS has been well established. However, whether BMI confers independent risk from smoking for progression to SP disease has not been previously studied with large sample sizes. Several studies have reported smoking as a risk factor for conversion to SPMS, without confining the analysis to pre-disease behavior (13–15). BMI is an attribute that could be speculated to be either a consequence of, or interact with, cigarette smoking to confer risk of disease progression. The present study provides evidence of an effect of high BMI (>30) at age 20 in smokers associated with increased risk of progression to SPMS, in patients who developed the disease after this age.

A possible explanation for increased SP risk among low and high BMI individuals could be enrichment of smokers in these categories. Because smoking may act as an appetite suppressant, it is unclear if increased smoking is responsible for higher number of low BMI individuals. Our models indicated that although low BMI individuals had increased hazard ratios for SP development, this effect was not statistically significant when low BMI was isolated from obesity.

The main finding of this study that obesity (BMI > 30) at age 20 in pre-diagnosis individuals is associated with increased SP risk is congruent with recent literature on BMI association with the risk of MS development. Several studies have found more than twofold risk of MS development for high BMI individuals at similar ages (6, 7). These studies indicate that the risk does not appear to be affected by earlier childhood BMI and is independent from known genetic factors.

Genetic association to increased BMI has also been reported to increase MS risk. Using a Mendelian randomization approach, Mokry and colleagues constructed weighted effect scores based on single nucleotide polymorphisms found to influence BMI to determine the risk of BMI on MS development. They reported a 1 SD increase in genetically determined BMI increased MS risk by 41% (23). A similar study found increased BMI, determined by a genetic risk score of BMI variants, significantly increased the risk of MS development, using EIMS and GEMS patients as a validation cohort (24).

Previous studies on the association between BMI and disability and clinical progression of MS patients have given differing and partly contradictive results. No association has been found between BMI and oligocloncal band positivity (25), disease duration, and EDSS score (25, 26) in some studies. The association between current BMI and the Patients Determined Disease Steps score has also been inconclusive (27, 28). Tettey and colleagues have recently shown that high BMI in patients with clinically isolated syndrome is associated with subsequent relapse and higher annual change in disability (29). This is in agreement with our observation of significantly younger onset age in obese patients compared with those with normal and low BMI (Table 1). High BMI (>25) has also been associated with poorer response to interferon-beta treatment (30). A significantly greater proportion of obese patients in our sample were exposed to immunomodulatory treatments (Table 1) which in fact indicates a more severe disease. However, despite this and correction for onset age, the risk of conversion to SPMS was still significantly increased in obese smokers and not in obese non-smokers, which would also be expected with systematic bias.

The mechanisms by which high BMI might increase the risk of MS or the risk of SP are unknown. High BMI is a potential driver of low-grade chronic inflammation. In particular, the hormone leptin secreted by adipose tissue is associated with reduced numbers of T-regulatory cells in MS patients (31), suggesting a disease driving mechanism which could lead to the SP stage.

Another possible pathway involves possible reduced vitamin D levels in overweight individuals, which is relevant since vitamin D is associated not only with increased MS risk (32), but also with relapse rate (33). High BMI in children (34) and adults (35) has been associated to lower 25-hydroxyvitamin D, an intermediary metabolite in the vitamin D metabolic pathway. It has been speculated that high BMI association with MS risk may in fact be a proxy for risk conferred by reduced vitamin D levels (6). While this cannot be ruled out, our study provides evidence that the increased risk of SP associated with obesity is confined to smokers, suggesting an interaction between the causative factor and smoking as opposed to simply the presence of confounding.

Body mass index has also been significantly associated to earlier disease onset (36). Although not the primary focus of the present study, we observed a similar correlation, with each BMI point resulting in 0.26 years reduced onset age in a simple linear regression.

One limitation of this study is while the data collection for EIMS and GEMS, as well as the SMSreg, is extensive, there is incomplete information. Therefore, we cannot rule out that factors not currently measured (such as socioeconomic status and initial disability level at the time of disease onset) could contribute to confounding. It also relies on self-reported information regarding both BMI and cigarette smoking, both of which may be prone to recall bias. It has previously been shown that those who are more overweight tend to under report their current weight (37). Such measurement error, if present, could potentially bias the association toward the null. A moderate correlation (*r* = 0.524) was observed between BMI at age 20 and at time of the questionnaire, reflecting the nearly 30-year average time lapse. Additionally, we were unable to include BMI after disease onset since (1) these data were not available except at the time of the questionnaire and (2) using such information includes a methodological dilemma, as whether high BMI or reduced mobility leads to the other is often unclear. Although historical BMI data are sparse in the SMSreg, tracking BMI over clinical course could yield information as to whether BMI during disease affects progression, and if a systematic weight control regiment for MS patient would be beneficial. Careful attention must be given to the potential confounding of disease severity on body composition and also the major confounding effect of smoking. Strengths of this study are longitudinal follow-up, large sample size, and using a population-based cohort.

The number of genetic and environmental factors which increase the risk of MS development is plentiful, while very few risk factors for MS progression have been identified. This presents a dilemma, since the triggering of MS and disease drivers could be different mechanisms. The lack of progress in detecting severity genes in MS, despite the success of determining risk genes, highlights this issue (38). However, this also presents an opportunity to identify risk factors of progression that can elucidate disease driving mechanisms. Such as in the case of smoking, BMI has been identified as a factor which not only increases the risk of developing MS but also of progressing to the SP stage, thereby offering clues as to the disease driving process.

## Author Contributions

AM and RR analyzed data and wrote the manuscript. AH collected and analyzed data. LA and TO collected data. JH collected data and wrote the manuscript.

## Conflict of Interest Statement

JH has received honoraria for serving on advisory boards for Biogen and Novartis and speaker’s fees from Biogen, Merck-Serono, Bayer-Schering, Teva, and Sanofi-Genzyme. He has served as the principle investigator for projects sponsored by, or has received unrestricted research support from, Biogen, Sanofi-Genzyme, Merck-Serono, TEVA, Novartis, and Bayer-Schering. TO received compensation for serving on scientific advisory boards, or conducting lectures, for Biogen and Genzyme as well as unrestricted research grants from Biogen, Bovartis, Genzyme, Allmiral, and AstraZeneca. The remaining coauthors declare that the research was conducted in the absence of any commercial or financial relationships that could be construed as a potential conflict of interest.
